# Antimicrobial Activity of *Terminalia catappa*, *Manilkara zapota* and *Piper betel* Leaf Extract

**DOI:** 10.4103/0250-474X.43012

**Published:** 2008

**Authors:** R. Nair, Sumitra Chanda

**Affiliations:** Phytochemical, Pharmacological and Microbiological Laboratory, Department of Biosciences, Saurashtra University, Rajkot-360 005, India

**Keywords:** *Terminalia catappa*, *Manilkara zapota*, *Piper betel*, antimicrobial activity, methanol extract

## Abstract

Aqueous and methanol extract of the leaves of *Terminalia catappa* L., *Manilkara zapota* L. and *Piper betel* L. were evaluated for antibacterial activity against 10 Gram positive, 12 Gram negative bacteria and one fungal strain, *Candida tropicalis*. Piperacillin and gentamicin were used as standards for antibacterial assay, while fluconazole was used as standard for antifungal assay. The three plants showed different degree of activity against the microorganisms investigated. The methanolic extract was considerably more effective than aqueous extract in inhibiting the investigated microbial strains. The most active antimicrobial plant was *Piper betel*.

There is a continuous and urgent need to discover new antimicrobial compounds with diverse chemical structures and novel mechanisms of action because there has been an alarming increase in the incidence of new and re-emerging infectious diseases. Another big concern is the development of resistance to the antibiotics in current clinical use 1–6.

*Terminalia catappa* L. belongs to the family Combretaceae. It is a tree and extensively planted in tropical India and Burma. The bark is rich in tannins, fruits are in ascorbic acid and seeds contain oil. The fruit is bitter, acrid, astringent and aphrodisiac. The leaves are maturant and emollient; the juice of leaves is used in the preparation of ointment for scabies, leprosy and other cutaneous diseases. The fruit is useful in bronchitis and bowels. The root bark is given in dysentery and diarrhea^7^. The fruits are used as antidiabetic, roots show antimicrobial activity. *Manilkara zapota* L. belongs to the family Sapotaceae. The plant is evergreen, glabrous tree with a milky juice. It is cultivated throughout India. The seeds are aperients, diuretic, tonic and febrifuge. The bark is antibiotic, astringent and febrifuge. It is used as tonic and the decoction is given in diarrhea and peludism^7^. *Piper betel* L. belongs to the family Piperaceae. This family usually contains herbs or shrubs often with swollen nodes, usually aromatic. The leaf is pungent, bitter, sweetish, acrid, heating, carminative, stomachic, anthelmintic, tonic, aphrodisiac and laxative. The leaves are useful in cough, foul smell in the mouth, ozoena, bronchitis, elephantiasis of the leg; improves appetite, it improves taste and appetite, tonic to the brain, heart, liver, strengthens the teeth, lessens thirst, clears the throat, vulnerary and styptic. The juice of the leaves is dropped into the eye in night blindness. The essential oil from the leaves is used in the treatment of catarrhal disorders and as an antiseptic. The decoction of leaves used for heating wounds. The fruit is employed with honey as a remedy for cough. The chemical constituents isolated are β-sitosterol^7^.

The present study reports the antibacterial activity of aqueous and ethanol extract of *T. cattappa, M. zapota* and *P. betel* leaf extracts against few clinically important gram positive and gram negative bacteria.

Fresh leaves of *Terminalia catappa* L., *Manilkara zapota* L. and *Piper betel* L. were collected randomly from the semi-arid region of Rajkot Gujarat, India in August 2003. The taxonomic identities of this plant were determined in the Department of Biosciences, Saurashtra University, Rajkot, Gujarat, India. Their voucher numbers are PSN 291, PSN 429 and SU/BIO/Thakrar 496, respectively. The plant material were washed under running tap water, air dried and then homogenized to fine powder and stored in airtight bottles.

Dried plant material (10 g) was extracted in distilled water (about 250 ml) for 6 h at slow heat. After every two hours it was filtered through eight layers of muslin cloth and centrifuged at 5000 g for 15 min. The supernatant was collected. This procedure was repeated twice and after 6 h, the supernatant was concentrated to make the final volume one-fifth of the original volume (50 ml). The extract was then autoclaved at 121° and 15 lbs pressure and stored at 4°.

Dried plant material (10g) was extracted with 100 ml of ethanol/methanol kept on a rotary shaker for 24 h. Thereafter, it was filtered and centrifuged at 5000 g for 15 min. The supernatant was collected and the solvent was evaporated to make the final volume one-fifth of the original volume. It was stored at 4° in airtight bottles for further studies.

The bacterial strains studied are identified strains and were obtained from National Chemical Laboratory (NCL), Pune, India. The studied microorganisms are *Pseudomonas aeruginosa* ATCC 27853, *P. testosteroni* NCIM5098, *P. pseudoalcaligenes* ATCC 17440, *Staphylococcus aureus* ATCC25923, *S. epidermidis* ATCC12228, *S. subflava* NCIM2178, *Proteus mirabilis* NCIM 2241, *P. vulgaris* NCTC8313, *P. morganii* NCIM2040, *Bacillus cereus* ATCC 11778, *B. subtilis* ATCC6633, *B. megaterium* ATCC9885, *Citrobacter freundii* ATCC10787, *Micrococcus flavus* ATCC10240, *Alcaligenes fecalis* ATCC 8750, *Enterobacter aerogenes* ATCC13048, *Salmonella typhimurium* ATCC23564, *Klebsiella pneumoniae* NCIM 2719, *Escherichia coli* ATCC25922, *Streptococcus fecalis* ATCC29212, *St. cremoris* NCIM2179, *St. agalactiae* NCIM 2401, and *Candida tropicalis* ATCC4563.

The antibacterial assay for screening aqueous extract of different plant species was evaluated by the method of agar disk diffusion method^8^. The media (Mueller Hinton Agar No. 2 and MRS media and Sabroaud dextrose agar for fungal strain) and the test bacterial cultures were poured into Petri dishes (Hi-Media). The test strain (200 μl) was inoculated into the media (inoculum size 10^ 8^ cells/ml) when the temperature reached 40-42°. The test compound (100 μl) was impregnated in to sterile discs (7 mm) (Hi-Media) and was then allowed to dry. The disc was then introduced into medium with the bacteria. The plates were incubated overnight at 37°. Microbial growth was determined by measuring the diameter of the zone of inhibition. The experiment was performed in triplicate and the mean values of the result are shown in Table 1.

**TABLE 1:** ANTIMICROBIAL ACTIVITY OF *TERMINALIA CATAPPA*, *MANILKARA ZAPOTA* AND *PIPER BETEL* LEAF EXTRACT

Microorganisms	*T. catappa*		*M. zapota*		*P. betel*		Standard		
				
	Aq	Me	Aq	Me	Aq	Me	G	Pc	Fu
*S. aureus*	5	-	5	10	-	13	-	32	-
*S. epidermidis*	-	-	4	10	-	7	22	11	-
*S. subflava*	-	12	1	6	-	7	-	20	-
*B. cereus*	-	5	7	6	6	13	20	13	-
*B. subtilis*	-	-	-	9	-	6	18	18	-
*B. megatarium*	5	9	3	6	-	6	31	13	-
*M. flavus*	8	-	4	16	4	14	23	25	-
*St. fecalis*	12	-	-	-	-	1	-	-	-
*St. cremoris*	2	-	-	-	-	4	-	-	-
*St. agalactiae*	-	-	-	-	-	3	-	-	-
*P. testosteroni*	-	11	-	4	2	10	28	-	-
*P. pseudoalcaligenes*	10	18	5	15	-	10	26	20	-
*P. mirabilis*	-	5	3	7	-	8	23	-	-
*P. vulgaris*	-	11	-	4	-	5	18	-	-
*P. morganii*	-	14	-	6	-	8	22	23	-
*A. fecalis*	-	15	-	5	4	10	21	-	-
*E. aerogenes*	-	1	-	11	-	9	20	22	-
*S. typhimurium*	-	10	-	3	-	7	27	20	-
*K. pneumoniae*	-	6	5	11	3	16	-	25	-
*E. coli*	-	-	-	5	-	12	26	14	-
*P. aeruginosa*	-	-	-	3	15	5	19	-	-
*C. freundii*	6	13	-	8	-	10	-	-	-
*C. tropicalis*	-	-	-	-	-	7	-	-	8

Antimicrobial activity of *Terminalia catappa*, *Manilkara zapota* and *Piper betel* leaf extract (aqueous and methanol) using agar disc diffusion method. The values are mean values of triplicate. G- gentamicin (10 μg/disc), Pc- piperacillin (100 μg/disc), Fu- fluconazole (10 μg/disc) Aq-aqueous, Me-methanol, - means no activity

The antibacterial assay for screening alcoholic extracts of different plant species was evaluated by the method of agar well diffusion method^9^–^10^. The media (Mueller Hinton Agar No. 2 and MRS media for bacteria and Sabroaud agar for fungal strain) and the test bacterial cultures were poured into Petri dishes (Hi-Media). The test strain (200 μl) was inoculated into the media (inoculum size 10^8^ cells/ml) when the temperature reached 40-42°. Care was taken to ensure proper homogenization. After the medium was solidified; a ditch was made in the plates with the help of a cup-borer (8.5 mm). The test compound (100 μl) was introduced into the well and the plates were incubated overnight at 37°. The experiment was performed under strict aseptic conditions. Inhibition of microbial growth was determined by measuring the diameter of the zone of inhibition. The experiment was performed in triplicates and the mean values of the result are shown in Table 1.

In general, Gram negative bacteria have been found to be more resistant than Gram positive bacteria^11^–^12^ but our results are in contrast to these previous reports. From the present work, it can be stated that methanolic extracts were more potent than aqueous extract. There are two possible explanations for the observed effects: i) the same active substances were present in water extracts but at concentrations at which bioactivity was no longer detectable and ii) active substances were soluble in organic solvents and therefore basically not present in water extracts^13^. *Streptococcus species* were most resistant to all the extracts except it exhibited very less activity for methanol extract of *Piper betel*. In addition all the extracts could not exhibit anticandidal activity against *C. tropicalis* except methanol extract of *Piper betel*. The methanolic extract of *P. betel* appears to be the most promising candidate for further work on isolation and identification of active compounds, in part because it appears to be active against a broader range of microbial species. However, the methanol extract of other two plant species also should be given adequate attention since even the inhibition of a single or few microbial species might reveal unexpected properties when the active compound(s) are isolated and purified. These findings indicate that further studies on chemical and biological properties of their active components should be performed.
